# Evidence of seasonality in the diagnosis of monocytic leukaemia

**DOI:** 10.1038/sj.bjc.6600497

**Published:** 2002-08-27

**Authors:** J P Eatough

**Affiliations:** Medical Physics Department, North Staffordshire Royal Infirmary, Princes Road, Hartshill, Stoke-on-Trent, ST4 7LN, UK

**Keywords:** monocytic leukaemia, seasonality, epidemiology, infection

## Abstract

Evidence of seasonality in the diagnosis of monocytic leukaemia in England and Wales is presented, with a maximum diagnosis rate in February/March and a minimum in August/September. Previous published results for monocytic leukaemia are of small sample size yet appear consistent with this finding.

*British Journal of Cancer* (2002) **87**, 509–510. doi:10.1038/sj.bjc.6600497 www.bjcancer.com

© 2002 Cancer Research UK

## 

There have been numerous studies of the possible seasonality of leukaemia diagnosis, however the literature fails to provide clear conclusions (Douglas *et al*, 1999). Many recent studies have focused on acute lymphocytic leukaemia and/or leukaemia in childhood but the results remain inconsistent (Harris *et al*, 1987; Badrinath *et al*, 1997; Westerbeek *et al*, 1998; Ross *et al*, 1999; Timonen, 1999; Higgins *et al*, 2001).

Fewer recent publications assess the possible seasonality of the other leukaemia subtypes in adults (Walker and van Noord, 1982; Douglas *et al*, 1999). Of particular relevance to this present work is the study by Walker and van Noord (1982) who analysed 7000 cases of acute leukaemia from the Third National Cancer Survey (TNCS), the Surveillance, Epidemiology and End Results programme (SEER) and the Central Cancer Patient Data System (CCPDS) in the United States. Overall, no evidence of seasonality was observed, and earlier positive reports from smaller studies (for example Hayes, 1961; Lee, 1963a, b; Gunz and Spears, 1968) were postulated to have been specious, possibly arising due to increased publication of positive results over negative findings. In fact Walker and van Noord did observe a significant seasonal variation for acute monocytic leukaemia within the TNCS and SEER data. This hypothesis was tested against the second smaller (CCPDS) data set, and lack of evidence of similar seasonality for monocytic leukaemia in this subsequent test led to the negative conclusion by the authors. However monocytic leukaemia is a rare disease and both data sets contained comparatively few cases.

The purpose of this paper is to re-examine the possibility of seasonality in the diagnosis of monocytic leukaemia, using a substantially larger data set.

## METHODS

Data were obtained from the Office of National Statistics (ONS) comprising the date of diagnosis for every recorded case of monocytic leukaemia in England and Wales during the period 1974–1998, amounting to 2311 cases. The number of diagnoses per calendar month was tested for evidence of a seasonal variation using the method of Walter and Elwood (1975) which is a refinement of the method developed by Edwards (1961). The method also allows a cosinor curve to be fitted to the data, with the maximum diagnosis rate of (1+α) times greater than the mean value, lying in the direction θ^*^. Other data sets available in the literature were similarly assessed as described below.

## RESULTS

Results obtained from the ONS data are given in Table 1

**Table 1:**
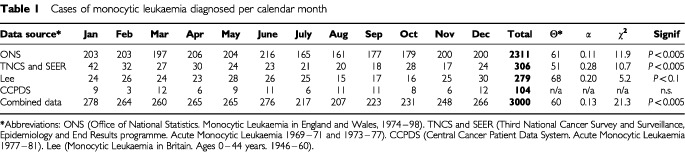
Cases of monocytic leukaemia diagnosed per calendar month

, and are shown normalised to months of equal length in Figure 1

**Figure 1:**
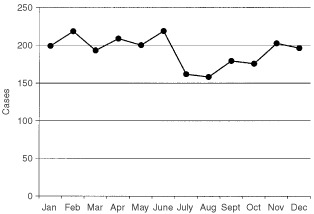
Cases of monocytic leukaemia diagnosed per calendar month in England and Wales 1974–1998

. The test statistic indicates a significant seasonal variation (χ^2=^11.9, *P*<0.005). The diagnosis rate shows a February/March maximum and an August/September minimum, with a peak-to-mean amplitude of 11%. The diagnosis rate decreases over the data collection period by around 1.5% per year on average, but correcting the monthly rates for this expected linear trend has little effect on either the significance of the observed seasonality (χ^2=^10.9, *P*<0.005), or on the amplitude and direction of the maximum (θ^*^= 60°, α=0.11).

Also shown in Table 1 are the two data sets analysed by Walker and van Noord (1982) and additional data presented by Lee (1963a). The TNCS and SEER data comprise 306 cases, and the test statistic indicates a significant seasonal variation (χ^2^ =10.7, *P*<0.005) with the fitted curve yielding a peak diagnosis rate at the end of February and an amplitude of 28%. Lee's data comprise month of clinical onset of symptoms for 279 cases, and while the test result for seasonality is not significant (*P*<0.1) the data again appear consistent with a late winter maximum and a summer minimum. Only the smallest (CCPDS) data set shows no evidence of a seasonal trend. Combining the data yields findings similar to those of the three largest individual data sets, but the test statistic is improved (χ^2^=21.3, *P*<<0.005).

## DISCUSSION

Preliminary modelling beyond that which is reported here suggests that the smallest data set (CCPDS) shown in Table 1, with around 100 cases, is insufficiently large to reliable determine the presence or otherwise of an underlying seasonal variation similar in magnitude to the other results. For the three remaining data sets there is broad consistency; in the level of significance of the test result (given the number of cases); in the apparent amplitude of the observed seasonality; and in the timing of the maximum and minimum rates of diagnosis with a February/March maximum and an August/September minimum. Analysis of further data that may be available through national cancer registries would be of interest to establish whether this finding is the result of chance or is more widely reproducible.

### Conclusion

Preliminary evidence of a seasonal variation in the diagnosis of monocytic leukaemia has been presented. Although a rare disease, confirmation of seasonality in the diagnosis of this leukaemia sub-type would represent evidence for infective or other causative or preventive etiological factors.
